# Longitudinal Impact of Disordered Eating Attitudes on Depression, Anxiety, and Somatization in Young Women with Anorexia and Bulimia

**DOI:** 10.3390/jcm13175078

**Published:** 2024-08-27

**Authors:** Sebastian Binyamin Skalski-Bednarz, Andreas Hillert, Janusz Surzykiewicz, Elisabeth Riedl, Jean-Pierre Harder, Sophia Magdalena Hillert, Marta Adamczyk, Patrycja Uram, Karol Konaszewski, Martin Rydygel, Kathrin Maier, Paweł Dobrakowski

**Affiliations:** 1 Faculty of Philosophy and Education, Catholic University of Eichstätt-Ingolstadt, 85072 Eichstätt, Germany; janusz.surzykiewicz@ku.de (J.S.); elisabeth.riedl@ku.de (E.R.); jean-pierre.harder@ku.de (J.-P.H.); sophia.hillert@ku.de (S.M.H.); 2 Institute of Psychology, Humanitas University, 41200 Sosnowiec, Poland; marta.adamczyk@humanitas.edu.pl (M.A.); paweldobrakowski@interia.pl (P.D.); 3 Specialist Center for Psychosomatics and Psychotherapy, Schön Klinik Roseneck, 83209 Prien am Chiemsee, Germany; ahillert@schoen-klinik.de; 4 Faculty of Education, Cardinal Stefan Wyszyński University in Warsaw, 01938 Warsaw, Poland; 5 Institute of Psychology, Polish Academy of Sciences, 00378 Warsaw, Poland; patrycja.uram@sd.psych.pan.pl; 6 Faculty of Education, University of Białystok, 15328 Białystok, Poland; k.konaszewski@uwb.edu.pl (K.K.); m.rydygel@uwb.edu.pl (M.R.); 7 Department of Educational Psychology in Social Work, Catholic University of Applied Sciences Munich, 80335 Munich, Germany; kathrin.maier@ksh-m.de

**Keywords:** eating disorders, anorexia, bulimia, EDI, disordered eating attitudes

## Abstract

**Background/Objectives:** Eating disorders, characterized by severe disturbances in eating behavior, are a public health concern, particularly among young women. This longitudinal study aimed to investigate the relationship between disordered eating attitudes and mental health outcomes, including depression, anxiety, and somatization, among individuals with eating disorders. **Methods:** A total of 471 young German women hospitalized with anorexia nervosa or bulimia nervosa participated in this study. Assessments were conducted at two points over a two-month interval using the Eating Disorder Inventory-2 (EDI-2) and the Patient Health Questionnaire. Regression analyses were performed to determine predictors of depression, anxiety, and somatization. The study controlled for socio-demographic segmentation using Sinus-Milieus, a social classification system, as a covariate. **Results:** Regression analyses revealed that interpersonal distrust and impulse regulation were significant predictors of subsequent depression. Impulse regulation strongly predicted subsequent anxiety, while both impulse regulation and social insecurity were strong predictors of subsequent somatization. The Neo-Ecological Milieu emerged as the most prevalent socio-demographic group (34.4% of participants). The Sinus-Milieus segmentation, however, did not significantly impact the health outcomes, suggesting consistent mental health issues across socio-demographic groups. **Conclusions:** This study’s findings underscored the role of disordered eating attitudes in predicting mental health outcomes among young women with eating disorders. Addressing these attitudes in therapeutic settings may help mitigate symptoms of not only the primary condition but also depression, anxiety, and somatization.

## 1. Introduction

Eating disorders represent a significant public health challenge, particularly impacting young individuals [1,2]. Marked by severe disturbances in eating behaviors, these disorders adversely affect both physical and mental health [3]. Among the various types of eating disorders, anorexia nervosa and bulimia nervosa are notably prevalent among adolescents and young adults [4]. This paper aims to examine these conditions by focusing on the psychological factors, particularly disordered eating attitudes, that contribute to their development and impact on mental health outcomes.

In developed countries, eating disorders are particularly prevalent among young people, with point prevalence rates of DSM-5 eating disorders reaching up to 15% in women and 3% in men [5]. These disorders often develop during adolescence or early adulthood, a critical period marked by significant physical, emotional, and social changes. The median age of onset for anorexia nervosa and bulimia nervosa is typically around 18 years [6], a time when individuals are particularly vulnerable to societal pressures related to body image and performance. The early identification and treatment of these disorders are crucial to prevent long-term physical and psychological consequences, as eating disorders can lead to severe malnutrition, cardiovascular issues, and persistent mental health problems [7].

Anorexia nervosa is characterized by an intense fear of gaining weight and a distorted body image, leading to restrictive eating behaviors and significant weight loss [8]. Individuals with anorexia nervosa often engage in extreme dieting, excessive exercise, and sometimes purging behaviors to avoid weight gain [9]. These behaviors result in severe physical health issues such as malnutrition, bone density loss, and cardiovascular problems. The psychological effects are equally severe, including high levels of anxiety, depression, and a distorted perception of body image, which perpetuate a cycle of unhealthy behaviors and thoughts [10].

Conversely, bulimia nervosa is characterized by recurrent episodes of binge eating followed by compensatory behaviors like vomiting, fasting, or excessive exercise to prevent weight gain [11]. Unlike anorexia nervosa, individuals with bulimia nervosa often maintain a normal weight, making the disorder less noticeable but equally harmful. The physical consequences of bulimia nervosa include electrolyte imbalances, gastrointestinal problems, and dental erosion due to frequent vomiting. Psychologically, bulimia nervosa is associated with intense feelings of guilt, shame, and a sense of loss of control during binge episodes, perpetuating the cycle of binging and purging [12].

Genetic factors play a crucial role in the development of eating disorders, with studies showing that the heritability of anorexia nervosa ranges from 33% to 84%, and for bulimia nervosa, from 28% to 83% [13]. Genome-wide association studies and other genetic analyses have identified several genetic loci associated with these disorders. Genetic variants in serotonergic and dopaminergic pathways have been linked to both anorexia nervosa and bulimia nervosa, influencing mood, reward, and feeding behaviors [14,15]. Additionally, alterations in neuropeptide Y and agouti-related peptide levels have been associated with increased orexigenic signaling in anorexia nervosa, while abnormalities in the secretion of peptides such as ghrelin, leptin, and cholecystokinin have been observed in bulimia nervosa [16,17].

The relationships between eating disorders and comorbid conditions like depression and anxiety are complex and multifaceted. Anxiety disorders and depression are among the most prevalent mental disorders in adolescence and are the most common comorbid diagnoses in eating disorders [18]. Studies have shown that individuals with lifetime major depressive disorder or anxiety disorder are significantly more likely to have a lifetime eating disorder [19]. Like anxiety disorders, individuals with eating disorders use dysfunctional strategies such as disordered eating to cope with their emotions [20]. Persisting comorbid anxiety symptoms after eating disorder remission increase the risk of relapse as individuals revert to former eating disorder-related coping behaviors [21]. In female adolescents and young adults, comorbid anxiety disorders are associated with greater eating disorder psychopathology [22]. Specifically, comorbid social anxiety has appeared to hamper recovery efforts as avoidance of interpersonal situations and fears of negative evaluations can interfere with treatment engagement and building a good therapeutic relationship [23].

Increasing prevalence rates have been reported for depressive disorders in adolescents and young adults, especially for girls [24]. Concerns about and negative perceptions of oneself and one’s appearance are associated with the onset and maintenance of eating disorders and depression [25]. Poor therapeutic outcomes may be explained by symptoms that reinforce each other: Dysfunctional social interaction patterns in depression lead to negative interpersonal experiences that increase the risk of dysfunctional emotion regulation (i.e., disordered eating) [26]. Eating pathology increases with greater severity of affective symptoms [27], and depressed mood improves with weight restoration [28].

Disordered eating attitudes, as measured using tools like the Eating Disorder Inventory (EDI) [29], have been extensively used to assess the severity of these conditions. The EDI evaluates various attitudinal and behavioral dimensions common in eating disorders, such as drive for thinness, bulimia nervosa, and body dissatisfaction, along with psychological traits like perfectionism, interpersonal distrust, and interoceptive awareness [30,31]. Given that these attitudes are deeply intertwined with emotion regulation and self-perception, they can also predict broader mental health issues [32]. Earlier studies like Segal and Golan’s [33] explored the cross-sectional correlations between total EDI scores and depression. These investigations revealed a moderate positive correlation, indicating that higher levels of disordered eating attitudes are linked to increased depressive symptoms. Additionally, Giovanni et al. [34] identified differences in EDI results between patients with eating disorders who have depression and those who do not, showing that patients with comorbid depression exhibited significantly higher levels of disordered eating attitudes.

Scores on scales related to body dissatisfaction and drive for thinness on the EDI reflect not only a preoccupation with weight and shape but also deeper issues of self-worth and identity [35,36,37], which are common in depression and anxiety [38,39,40]. Perfectionism and interpersonal distrust, frequently observed in individuals with eating disorders, can contribute to chronic stress and social isolation, exacerbating anxiety and depressive symptoms [41,42] (e.g., through persistent negative thinking [43]). Additionally, interoceptive awareness deficits, reflecting difficulties in recognizing and responding to internal emotional states, are linked with both eating disorders and somatization [44], where psychological distress manifests as physical symptoms [45]. Thus, the attitudinal measures used to gauge eating disorder severity could also serve as indicators for broader psychopathological risk. Addressing these attitudes in treatment could therefore provide dual benefits: reduce eating disorder symptoms and mitigate the risk of comorbid mental health conditions. By integrating psychological and behavioral interventions that address these underlying attitudes, clinicians might better support patients in achieving holistic and sustainable recovery.

### Current Study

The aim of this longitudinal study was to examine the relationships between eating disorder attitudes and mental health outcomes, including depression, anxiety, and somatization, among young women hospitalized with anorexia nervosa or bulimia nervosa. By assessing these relationships over a two-month interval, this study sought to identify key psychological factors that predict negative mental health outcomes in this population. Additionally, the study controlled for socio-demographic segments, categorized using Sinus-Milieus (see Section 2.2.3), to provide a more comprehensive understanding of how different social and lifestyle environments may shape relationships between disordered eating attitudes and comorbid mental health conditions.

## 2. Materials and Methods

### 2.1. Participants and Procedure

This two-wave longitudinal study, approved by a university ethics committee, was conducted over a two-month period in 2023. Data were collected from individuals diagnosed with eating disorders who were receiving care at Schön Klinik in Bavaria, Germany. All participants were hospitalized with a diagnosis of eating disorders (F50, according to the 11th edition of the International Classification of Diseases [46]). The first wave of data collection occurred upon participants’ admission to the clinic, while the second wave took place during a follow-up visit after discharge. It is important to note that the study did not control for therapeutic interventions during hospitalization. As a result, it does not assess within-person changes in mental health variables. Instead, the study focuses on whether baseline data can predict changes in the dependent variable, accounting for the autoregressive effects of the initial measurement.

During the study period, patients participated in therapy sessions focused on addressing unhealthy relationships with food, weight, and body image. These sessions involved professional support aimed at changing thoughts and behaviors related to eating disorders. Participants engaged in counseling sessions designed to help them express their emotions and develop healthier coping mechanisms. The therapy aimed to provide early support, helping participants gain control over their eating disorders rather than being controlled by them.

Each participant provided informed consent and completed anonymous paper-and-pencil questionnaires during their appointments with the attending physician. In the first wave (T1), participants completed surveys measuring disordered eating attitudes, mental health (including depression, anxiety, and somatization), and socio-demographic conditions. In the second wave (T2), the participants completed surveys assessing depression, anxiety, and somatization. To ensure anonymity, participants signed both surveys using an invented ID that did not identify them personally. The retention rate for the study was 83%.

All participants were female and diagnosed with either anorexia nervosa or bulimia nervosa. Although sex was not a criterion for recruitment, only three males responded to the survey. Due to the unrepresentative nature of this small male sample, their data were excluded from the analysis. Additionally, initial data from two women diagnosed with other eating disorders were also excluded for the same reasons. The final sample consisted of 471 young women with a mean age of 23.56 (*SD* = 3.12) years.

**Figure 1:**
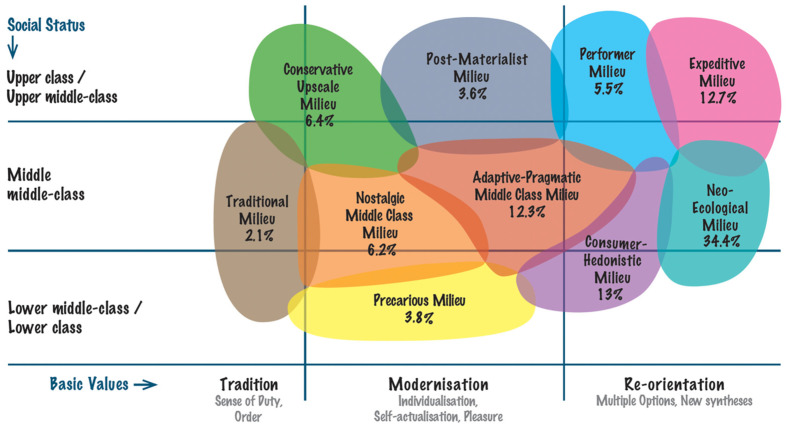
Characteristics of participants with eating disorders (total) across socio-demographic Sinus-Milieus segments.

The participant characteristics across various socio-demographic Sinus-Milieus segments are presented in Table 1 and Figure 1. The largest segment of participants fell into the Neo-Ecological Milieu category (34.4% for total, 34.3% for bulimia nervosa, and 34.5% for anorexia nervosa). The Consumer-Hedonistic Milieu was also highly represented (13% for total, 12.9% for bulimia nervosa, and 13.1% for anorexia nervosa). The smallest segment was the Traditional Milieu (2.1% for total, 2.2% for bulimia nervosa, and 2.0% for anorexia nervosa).

### 2.2. Measures

#### 2.2.1. Disordered Eating Attitudes

The Eating Disorder Inventory-2 (EDI-2) developed by Garner [29] and adapted into German by Thiel et al. (1997) was used to assess emotional, cognitive, and behavioral aspects of disordered eating attitudes. This self-report questionnaire measures 11 dimensions commonly linked to anorexia nervosa and bulimia nervosa on a 6-point response scale ranging from 1 (never) to 6 (always), with higher scores indicating greater symptomatology. Sample items include “I am terrified of gaining weight” (drive for thinness) and “I have gone on eating binges where I feel that I may not be able to stop” (bulimia nervosa). The subscales include: (1) drive for thinness: measures excessive concern with dieting, preoccupation with weight, and fear of weight gain; (2) bulimia: assesses the tendency to engage in episodes of binge eating followed by purging behaviors; (3) body dissatisfaction: evaluates discontent with overall body shape and specific parts of the body; (4) ineffectiveness: reflects feelings of general inadequacy, insecurity, worthlessness, and a lack of control over one’s life; (5) perfectionism: assesses the extent to which an individual has high personal standards and places significant importance on achieving perfection; (6) interpersonal distrust: measures reluctance to form close relationships and a general distrust of others; (7) interoceptive awareness: evaluates the ability to recognize and accurately identify internal bodily signals and emotions; (8) maturity fears: reflects a desire to return to the security of childhood and a reluctance to assume adult responsibilities; (9) asceticism: assesses the tendency to seek virtue through self-discipline, self-denial, and the control of bodily urges; (10) impulse regulation: evaluates the ability to manage impulsive behaviors and emotional responses; and (11) social insecurity: measures feelings of social alienation and perceived inadequacy in social interactions. The EDI-2 has been validated in various populations, demonstrating strong psychometric properties, including high internal consistency and test–retest reliability [29,30,31,47]. In this study, internal consistency for the subscales ranged from 0.73 to 0.85 (Cronbach’s alpha).

#### 2.2.2. Depression, Anxiety, and Somatization

The Patient Health Questionnaire (PHQ) in the German language [48] was utilized to screen mental health disorders based on the criteria of the Diagnostic and Statistical Manual of Mental Disorders (DSM). The PHQ is structured into modules that measure specific psychological disorders. For this study, we utilized the Generalized Anxiety Disorder (GAD), depression, and somatization modules [49]. The GAD module is comprised of seven items that assess the frequency of anxiety-related problems over the past 4 weeks. Response options are expressed on a 3-point scale, ranging from 0 (none) to 2 (more than half the days). The presence of GAD is defined by four or more responses of “more than half the days” (2), including a positive response to the item “feeling nervous, anxious, tense, or very worried”. The depression module (PHQ-9) evaluates the presence of symptoms over the past 2 weeks, such as “poor appetite or overeating” and “feeling bad about yourself or that you are a failure or have let yourself or your family down” [50]. It includes nine items with 4-point response categories: 0 (never), 1 (less than a week), 2 (a week or more), and 3 (almost every day). The sum of the responses is used to calculate the total score, with a score of ≥10 being the recommended cut-off point for diagnosing depression. The somatization module (PHQ-15) assesses somatic symptom severity to diagnose somatoform disorders [51]. This module surveys 15 somatic symptoms or symptom clusters, accounting for over 90% of physical complaints (excluding upper respiratory symptoms) reported in outpatient settings. For 13 of the symptoms, participants are asked, “During the past 4 weeks, how much have you been bothered by any of the following problems?” The three scoring options are coded as 0 (not bothered at all), 1 (bothered a little), and 2 (bothered a lot), with a score of 2 indicating a severe somatic symptom. The PHQ-15 also includes two additional items related to physical symptoms: “feeling tired or having little energy” and “trouble sleeping”. The PHQ modules have been validated in various populations, demonstrating strong psychometric properties, including high internal consistency and test–retest reliability [52,53,54]. In this study, the internal consistency for the subscales, as measured using Cronbach’s alpha, ranged from 0.86 to 0.94.

#### 2.2.3. Sinus-Milieus

Each participant’s milieu was assessed using the standardized Sinus-Milieus multi-item scale developed by SINUS Marktforschung GmbH [55]. Responses to items were recorded on a 4-point response format ranging from 1 (does not apply at all) to 4 (applies completely). Sinus-Milieus is a social classification system that segments the population into distinct groups based on a combination of socio-demographic variables, lifestyles, and value orientations: (1) conservative upscale milieu: characterized by traditional values, a high degree of social responsibility, and a preference for order and stability (they often hold leadership positions and value exclusivity and status); (2) post-materialist milieu: representing the educated elite with post-materialistic values, this group emphasizes self-determination, sustainability, and social equality (they advocate for growth beyond materialism and are deeply committed to community welfare); (3) performer milieu: efficiency-oriented and optimistic about progress, this group comprises the economic elite who value self-reliance and global thinking (they are pioneers in style and consumption, with high technological and digital affinity); (4) expeditive milieu: ambitious, creative bohemians who are urban, digital, and cosmopolitan (they seek unconventional experiences and new boundaries, presenting themselves as post-modern elites); (5) traditional milieu: this group values security and order often rooted in the traditional working-class culture (they prefer simplicity and adapt to necessity with increasing acceptance of sustainability norms); (6) nostalgic middle class milieu: the harmony-oriented lower middle class, longing for secure conditions and appropriate status (they feel overwhelmed by societal changes and have a growing desire for the “good old days”); (7) adaptive-pragmatic middle class milieu: the modern mainstream, characterized by adaptability, pragmatism, and a desire for enjoyment and entertainment (they seek security and belonging but are increasingly discontented with societal developments); (8) neo-ecological milieu: progressive realists who are optimistic yet aware of global challenges (they see themselves as changemakers, balancing success with sustainability and openness to new value syntheses); (9) precarious milieu: this group strives for orientation and social inclusion, often facing social disadvantages and exclusion (they feel left behind and harbor resentment, viewing themselves as resilient survivors); and (10) consumer-hedonistic milieu: focused on consumption and entertainment, this group enjoys living in the moment with a strong need for recognition (they balance work adaptation with leisure escapism and resist the pressure of sustainability and political correctness). The Sinus-Milieus study is a commercial project by SINUS-Institut [sinus-institut.de]. Therefore, based on licensing agreements, we cannot provide detailed information on the specific questions. With this system, we classified each participant into one of these groups based on a set of questions.

### 2.3. Statistical Analyses

This study’s hypotheses were tested using multiple regression analyses, *t*-tests for independent samples, and one-way ANOVAs in SPSS (Version 29). In the regression analyses, each model included baseline attitudes related to eating disorders, the baseline value of either depression, anxiety, or somatization (autoregression), and sociodemographic covariates. The models were constructed for the total sample and separately for participants with anorexia nervosa or bulimia nervosa. The outcome variables were the subsequent measures of the same mental health indicators at T2 (depression, anxiety, or somatization). All variables were initially examined for potential outliers. Given the relatively normal distributions and the absence of significant outliers, original values were used for all variables. Statistical significance was determined at *p* ≤ 0.05, and effect sizes were assessed using *R*^2^ and Cohen’s *d*.

## 3. Results

Table 2 presents the descriptive statistics for participants with eating disorders, categorized into the total sample, bulimia nervosa, and anorexia nervosa groups. *T*-tests revealed that young women with bulimia nervosa had higher mean scores on the drive for thinness (at) T1 (*d* = −0.58), bulimia T1 (*d* = −1.54), impulse regulation T1 (*d* = −0.4), social insecurity T1 (*d* = −0.29), and somatization T2 (*d* = −0.31) compared to those with anorexia nervosa. One-way ANOVA results indicated no significant differences in depression, anxiety, and somatization at T1 and T2 (*p* > 0.05) across the socio-demographic Sinus-Milieus segments. However, the regression analyses reported in Table 3 identified several disordered eating attitudes as significant predictors of mental health among young women with anorexia nervosa and bulimia nervosa.

To ensure the accuracy of the predictors, we checked variance inflation factor (VIF) values to control for multicollinearity in the regression models. The VIF values, all below 5 except for autoregression, generally indicated low to moderate multicollinearity among the predictor variables.

Our regression analyses revealed significant effects of several variables on depression at T2 across different eating disorder groups, beyond autoregressive effects. For the total sample, significant predictors were interpersonal distrust at T1 (β = 0.14, *p* < 0.05) and impulse regulation at T1 (β = 0.18, *p* < 0.001). For individuals with bulimia nervosa, impulse regulation at T1 (β = 0.31, *p* < 0.001) was a significant predictor. Among individuals with anorexia nervosa, significant predictors were body dissatisfaction at T1 (β = 0.16, *p* < 0.05) and impulse regulation at T1 (β = −0.16, *p* < 0.05). The models for depression showed a good fit to the data: *F*_(14, 369)_ = 15, *p* < 0.001, *R*^2^ = 0.36 (total sample), *F*_(14, 97)_ = 5.5, *p* < 0.01, *R*^2^ = 0.44 (bulimia nervosa), and *F*_(14, 258)_ = 10.33, *R*^2^ = 0.36 (anorexia nervosa).

For anxiety at T2, the only significant predictor beyond autoregression in the total sample was impulse regulation at T1 (β = 0.23, *p* < 0.001). Among individuals with bulimia nervosa, the same significant effect was observed (β = 0.37, *p* < 0.001). A similar effect was noted for individuals with anorexia nervosa, where impulse regulation at T1 was also the only significant predictor (β = 0.19, *p* < 0.05). The model for anxiety also demonstrated a good fit to the data: *F*_(14, 369)_ = 16.52, *p* < 0.001, *R*^2^ = 0.39 (total sample), *F*_(14, 97)_ = 6.35, *p* < 0.001, *R*^2^ = 0.49 (bulimia nervosa), and *F*_(14, 258)_ = 11.45, *R*^2^ = 0.39 (anorexia nervosa).

Regarding somatization at T2, significant predictors, supplementary to the effect of somatization at T1, in the total sample included age (β = 0.1, *p* < 0.05), impulse regulation at T1 (β = 0.14, *p* < 0.05), and social insecurity at T1 (β = 0.15, *p* < 0.05). Among individuals with anorexia nervosa, significant predictors were age (β = 0.12, *p* < 0.05) and social insecurity at T1 (β = 0.17, *p* < 0.05). The somatization models also exhibited a good fit to the data: *F*_(14, 369)_ = 14.14, *p* < 0.001, *R*^2^ = 0.45 (total sample), *F*_(14, 97)_ = 4.7, *p* < 0.001, *R*^2^ = 0.4 (bulimia nervosa), and *F*_(14, 258)_ = 17.57, *R*^2^ = 0.49 (anorexia nervosa).

## 4. Discussion

This study investigated the longitudinal impact of disordered eating attitudes on depression, anxiety, and somatization in young women diagnosed with anorexia nervosa or bulimia nervosa. Analyzing data from 471 participants over a two-month period, we identified significant predictors of mental health outcomes based on specific disordered eating attitudes. Consistent with previous research [10,18,19,22], we found that individuals with eating disorders frequently experienced depression and difficulties in emotion regulation. Our findings extend this understanding by demonstrating that women with anorexia nervosa and bulimia nervosa can exhibit similar baseline levels of depression, anxiety, and somatization, reinforcing the conceptualization of eating disorders as a transdiagnostic syndrome [56,57].

These results indicate that specific disordered eating attitudes, particularly interpersonal distrust and impulse regulation, can be significant predictors of depression, anxiety, and somatization among women with eating disorders in Germany. For instance, interpersonal distrust and impulse regulation significantly predicted subsequent depression. Impulse regulation was also a significant predictor of subsequent anxiety, while both impulse regulation and social insecurity robustly predicted subsequent somatization. These findings aligned with prior research suggesting that interpersonal distrust can exacerbate feelings of isolation and hopelessness, thereby increasing the risk of depression and anxiety [41,42]. Similarly, difficulties in impulse regulation, often manifesting as impulsive behaviors, can lead to heightened somatic complaints due to the stress associated with a perceived loss of control [58].

Our findings are consistent with the study by Giovanni et al. [34], who identified impulse regulation and social insecurity as key differentiating factors between Italian participants with eating disorders who experienced depression and those who did not. Impulse regulation and social insecurity appeared to play a critical role in the comorbidity of eating disorders and depression in our study.

Moreover, our results aligned with the cross-sectional study by Segal and Golan [33], who linked overall disordered eating attitudes to increased levels of anxiety and emotional distress. Both studies indicated that individuals with dysfunctional thinking often resort to unhealthy coping mechanisms to temporarily alleviate negative emotions. Similarly, Aspen [59] found a strong association between eating disorder symptoms and anxiety disorders, mood disorders, and insomnia, with the severity and type of these comorbidities worsening as eating disorder symptoms intensified.

The current study revealed notable differences in the effects of disordered eating attitudes between bulimia and anorexia. Generally, impulse regulation emerged as a leading predictor of depression and anxiety across both types of eating disorders, highlighting the broad relevance of impulse control issues in the psychological profiles of these patients. However, the effects of somatization were more complex. Impulse regulation significantly predicted somatization for the entire sample, while social insecurity was a significant predictor specifically for anorexia. No significant predictors of somatization were identified for bulimia. Additionally, age was a significant predictor of somatization in the total sample and among those with anorexia, suggesting that older individuals may be more susceptible to these outcomes.

The lack of significant predictors for bulimia might be explained by the presence of comorbidities. For instance, Marsh et al. [60] highlighted reduced activation in the fronto-striatal region exclusively in bulimia, but not in anorexia, suggesting developmental differences, clinical severity, chronicity, and the presence of comorbid conditions in bulimia compared to other eating disorders. It appeared these comorbid conditions could overshadow the impact of disordered eating attitudes on somatization, thereby shaping the somatic symptoms in this group. In contrast, the significance of social insecurity in anorexia could reflect the heightened interpersonal sensitivity and isolation often observed in these patients, which tends to accumulate and intensify in the severe and enduring stages of anorexia [61].

The observation that young women with bulimia nervosa exhibited higher mean scores on the drive for thinness, bulimia, impulse regulation, and social insecurity compared to those with anorexia nervosa illuminated additional psychological nuances between these disorders. Individuals with bulimia nervosa often experience intense preoccupations with body image and a strong drive for thinness, leading to cyclical binge–purge behaviors as maladaptive coping mechanisms; these can result in a more intense absorption in the drive for thinness than in anorexia [3]. These behaviors can exacerbate difficulties in impulse regulation, reflecting a broader pattern of emotion dysregulation that complicates recovery [23]. Elevated social insecurity scores of bulimia nervosa patients may stem from the secretive and shame-inducing nature of binge–purge episodes, fostering feelings of social alienation and low self-worth [12]. Additionally, differences in subsequent somatization were observed; however, these changes appeared to be related to the specific treatments and conditions in the clinic, which were not controlled for in this study. Our focus was on the longitudinal causal effects between disordered eating attitudes and mental health, not on temporal changes in mental health, and we did not control for subject-specific manipulations or treatments.

Interestingly, this study’s use of the Sinus-Milieus segmentation revealed that socio-demographic factors did not significantly impact mental health outcomes, indicating consistent mental health challenges across different social groups. In Germany, the Post-Materialist Milieu, characterized by values of self-determination, social equality, and post-materialist principles, is typically the most popular group [62]. However, our study sample, comprised of predominantly younger individuals, primarily fell into the Neo-Ecological Milieu, a group noted for its progressive realism, environmental awareness, and openness to new value syntheses, reflecting a generational shift toward more ecological and sustainable perspectives among younger cohorts [63,64].

While the Sinus-Milieus approach provides valuable insights into segmenting participants based on socio-demographic variables, lifestyles, and value orientations, it may be beneficial to complement it with more traditional socio-demographic measures, such as standard sociological surveys, for a more comprehensive perspective. It is also important to note that Germany’s population is predominantly White Caucasian (with Whites and Hispanics exhibiting nearly three times the prevalence of depression compared to African-Americans; see [65]). Additionally, the study focused on young women who are likely still in the process of achieving economic and familial independence. This significant homogeneity of the sample precludes a reliable assessment of these variables as potential predictors.

### 4.1. Practical Implications

Our results suggest that therapeutic interventions targeting disordered eating attitudes, particularly impulse regulation and interpersonal distrust, could be crucial in mitigating not only the primary symptoms of eating disorders but also associated mental health conditions. Our findings underscored the pervasive nature of eating disorders and their related mental health issues, which transcend socio-demographic boundaries (e.g., the above Sinus-Milieus segmentation). This universality highlights the importance of developing inclusive therapy strategies that can effectively benefit individuals across diverse social groups. The elevated drive for thinness and social insecurity among bulimia nervosa patients emphasizes the need for tailored interventions that address these specific attitudes.

Specific therapeutic approaches like dialectical behavior therapy (DBT) and cognitive behavioral therapy (CBT) have shown efficacy in addressing these issues. DBT focuses on improving emotion regulation and impulse control through skills training in mindfulness, distress tolerance, emotion regulation, and interpersonal effectiveness [66]. This therapy is particularly beneficial for individuals with high levels of impulsivity and emotion dysregulation, which are common in bulimia nervosa.

CBT targets maladaptive thoughts and behaviors associated with eating disorders and comorbid depression and anxiety. By restructuring negative thought patterns and promoting healthier behaviors, CBT can effectively reduce symptoms of both eating disorders and their psychological comorbidities [67]. Additionally, integrating interpersonal psychotherapy can help address issues of interpersonal distrust by improving social functioning and relationships, thereby reducing social insecurity [68]. Furthermore, incorporating mindfulness-based interventions can help patients develop a non-judgmental awareness of their thoughts and feelings, reducing the impact of disordered eating attitudes and improving overall mental health outcomes [69]. Such comprehensive treatment strategies that address both the eating disorder and its psychological comorbidities can ultimately enhance the overall well-being of affected individuals.

### 4.2. Limitations

While this study provided valuable insights, several limitations should be noted. First, the sample was restricted to young women hospitalized with anorexia nervosa or bulimia nervosa, and the results may not generalize to all individuals with eating disorders, particularly those not requiring hospitalization or those with different eating disorders. Future research should include a more diverse sample to enhance the generalizability of the findings. Second, the study was conducted over a relatively short interval of 2 months. Longer-term studies are needed to understand the enduring impact of disordered eating attitudes on mental health. Furthermore, while the study controlled for socio-demographic variables using the Sinus-Milieus system, other factors like genetic predispositions and environmental influences were not accounted for and could play significant roles in the development and persistence of eating disorders and mental health comorbidities. Moreover, the patients participated in CBT and remained on pharmacotherapy during this study, which may have influenced their levels of depression, anxiety, and somatization. However, these treatments should not have interfered with the predictive effects between model terms, which was the primary focus of this research. In summary, while the study’s longitudinal design was an important advancement, incorporating more time points in future data collection efforts and controlling for additional confounding variables would be beneficial.

The limitations associated with evaluating socio-demographic groups warrant separate consideration. Although the Sinus-Milieus method effectively addresses issues related to interpretation variability and researcher biases—due to the automatic segmentation conducted by the SINUS-Institut—there are other potentially significant limitations to note. One such limitation is the static nature of these segments, which may not fully capture the dynamic and evolving nature of socio-demographic factors. Additionally, this categorization might oversimplify the complex and fluid nature of patients’ social identities and life experiences. Nevertheless, when combined with other methodologies, the Sinus-Milieus approach could enhance the comprehensive understanding of the diverse backgrounds of participants in future studies.

## 5. Conclusions

Overall, this study provided valuable insights into the predictive role of disordered eating attitudes on mental health outcomes in young women with anorexia nervosa and bulimia nervosa. Our findings underscored the substantial potential impact of specific psychological factors, such as impulse regulation and interpersonal distrust, not only on eating disorders but also on concurrent mental health outcomes. Addressing disordered eating attitudes in therapeutic settings can be a beneficial component in treating the primary condition and preventing the exacerbation of associated mental health issues. With these results, we recommend a holistic clinical approach that integrates psychological and behavioral interventions that target disordered eating attitudes to support comprehensive recovery and improve overall mental health.

## Figures and Tables

**Table 1:** Participant characteristics across socio-demographic Sinus-Milieus segments.

Sinus-Milieus Segment	Total (%)	Bulimia (%)	Anorexia (%)
Conservative Upscale Milieu	6.4	6.3	6.5
Post-Materialist Milieu	3.6	3.7	3.5
Performer Milieu	5.5	5.4	5.6
Expeditive Milieu	12.7	12.6	12.8
Traditional Milieu	2.1	2.2	2
Nostalgic Middle-Class Milieu	6.2	6.1	6.3
Adaptive-Pragmatic Middle-Class Milieu	12.3	12.2	12.4
Neo-Ecological Milieu	34.4	34.3	34.5
Precarious Milieu	3.8	3.9	3.7
Consumer-Hedonistic Milieu	13	12.9	13.1

**Table 2:** Descriptive statistics.

	**Eating Disorders (Total)**	**Bulimia** **(*N* = 134)**	**Anorexia** **(*N* = 337)**	**Anorexia vs. Bulimia**	
**Variable**	***M* (*SD*)**	***M* (*SD*)**	***M* (*SD*)**	***t***	***d***
Drive for Thinness T1	4.29 (1.29)	4.80 (1)	4.09 (1.33)	−5.61 ***	−0.58
Bulimia T1	2.84 (1.57)	4.25 (1.19)	2.26 (1.33)	−15.01 ***	−1.54
Body Dissatisfaction T1	4.38 (1.12)	4.83 (1.1)	4.19 (1.08)	−5.66	−0.58
Ineffectiveness T1	3.89 (1.04)	4.00 (0.95)	3.84 (1.06)	−1.52	−0.16
Perfectionism T1	3.81 (1.02)	3.68 (1.05)	3.86 (1)	1.71	0.18
Interpersonal Distrust T1	3.46 (0.92)	3.60 (0.9)	3.4 (0.93)	−2.12	−0.22
Interoceptive Awareness T1	3.76 (0.99)	4.11 (0.86)	3.62 (1)	−4.92	−0.51
Maturity Fears T1	3.43 (0.93)	3.39 (0.95)	3.44 (0.92)	0.53	0.06
Asceticism T1	3.28 (0.91)	3.32 (0.76)	3.26 (0.97)	−0.56	−0.06
Impulse Regulation T1	2.65 (0.78)	2.87 (0.71)	2.56 (0.79)	−3.87 ***	−0.4
Social Insecurity T1	3.60 (0.82)	3.77 (0.75)	3.53 (0.84)	−2.83 **	−0.29
Depression T1	15.59 (5.89)	12.39 (5.6)	11.84 (5.75)	0.07	0.01
Anxiety T1	10.94 (5.26)	15.55 (6.01)	15.60 (5.85)	0.58	0.06
Somatization T1	12.00 (5.71)	10.72 (5.16)	11.03 (5.3)	−0.94	−0.1
Depression T2	8.56 (5.82)	9.01 (5.38)	8.38 (5.99)	−0.97	−0.11
Anxiety T2	6.71 (4.78)	6.42 (4.36)	6.82 (4.94)	0.75	0.09
Somatization T2	8.26 (4.98)	9.36 (4.94)	7.82 (4.94)	−2.81 **	−0.31

** *p* < 0.01, *** *p* < 0.001.

**Table 3:** Disordered eating attitudes as predictors of subsequent negative impacts on mental health in young women with anorexia nervosa and bulimia nervosa.

	Eating Disorders (Total)		Bulimia (*N* = 134)		Anorexia (*N* = 337)	
Variable	*β*	*t*	*β*	*t*	*β*	*t*
Outcome: Depression T2						
Constant	--	−4.26 ***	--	−1.99 *	--	−4.03 ***
Age	0.01	0.05	−0.02	−0.22	0.04	0.64
Drive for Thinness T1	0.08	1.31	0.03	0.21	0.1	1.29
Bulimia T1	−0.01	−0.16	−0.07	−0.73	0.03	0.56
Body Dissatisfaction T1	0.07	1.12	−0.09	−0.76	0.16	2.07 *
Ineffectiveness T1	0.04	0.46	0.01	0.02	0.05	0.48
Perfectionism T1	0.06	1.21	0.06	0.69	0.05	0.86
Interpersonal Distrust T1	0.14	2.13 *	0.15	1.4	0.11	1.33
Interoceptive Awareness T1	−0.03	−0.43	0.06	0.45	−0.02	−0.23
Maturity Fears T1	0.07	1.45	0.03	0.36	0.08	1.39
Asceticism T1	−0.11	−1.77	0.05	0.45	0.13	1.65
Impulse Regulation T1	0.18	2.98 ***	0.31	3.08 ***	0.16	2.09 *
Social Insecurity T1	0.11	1.51	0.12	0.85	0.11	1.2
Depression T1	0.25	4.21 ***	0.3	3.01 ***	0.23	3.12 ***
Sinus-Milieus	−0.06	−1.32	−0.03	−0.41	−0.06	−1.08
	*F*_(14, 369)_ = 15 ***, *R*^2^ = 0.36		*F*_(14, 97)_ = 5.5 ***, *R*^2^ = 0.44		*F*_(14, 258)_ = 10.33 ***, *R*^2^ = 0.36	
Outcome: Anxiety T2						
Constant	--	−2.98 ***	--	−1.98 *	--	−2.93 ***
Age	−0.06	−1.24	0.01	0.04	−0.04	−0.74
Drive for Thinness T1	0.06	0.89	−0.05	−0.45	0.09	1.12
Bulimia T1	−0.08	−1.54	0.01	0.14	−0.01	−0.18
Body Dissatisfaction T1	0.09	1.41	0.1	0.82	0.14	1.82
Ineffectiveness T1	0.01	0.04	−0.17	−1.24	0.02	0.24
Perfectionism T1	0.01	0.28	0.02	0.27	0.01	0.22
Interpersonal Distrust T1	−0.01	-0.16	0.03	0.33	−0.03	−0.38
Interoceptive Awareness T1	0.03	0.45	0.05	0.43	0.06	0.7
Maturity Fears T1	0.08	1.7	0.02	0.25	0.1	1.73
Asceticism T1	−0.06	−0.99	−0.06	−0.58	−0.09	−1.12
Impulse Regulation T1	0.23	3.78 ***	0.37	3.57 ***	0.19	2.39 *
Social Insecurity T1	0.13	1.86	0.25	1.9	0.11	1.23
Anxiety T1	0.28	4.89 ***	0.33	3.25 ***	0.23	3.15 ***
Sinus-Milieus	−0.06	−1.38	−0.12	−1.48	−0.05	−0.85
	*F*_(14, 369)_ = 16.52 ***, *R*^2^ = 0.39		*F*_(14, 97)_ = 6.35 ***, *R*^2^ = 0.49		*F*_(14, 258)_ = 11.45 ***, *R*^2^ = 0.39	
Outcome: Somatization T2						
Constant	--	−1.99 *	--	−1.98 *	--	−1.99 *
Age	0.1	2.24 *	0.06	0.6	0.12	2.31 *
Drive for Thinness T1	−0.06	−1.03	−0.04	−0.28	−0.09	−1.31
Bulimia T1	−0.01	−0.18	−0.14	−1.4	−0.05	−0.97
Body Dissatisfaction T1	0.1	1.65	−0.02	−0.13	0.11	1.65
Ineffectiveness T1	−0.11	−1.56	−0.21	−1.45	−0.04	−0.43
Perfectionism T1	0.02	0.41	0.09	1.11	−0.02	−0.31
Interpersonal Distrust T1	0.02	0.41	0.14	1.2	−0.06	−0.88
Interoceptive Awareness T1	0.01	−0.07	0.04	0.28	0.02	0.27
Maturity Fears T1	0.05	1.19	0.01	−0.03	0.08	1.58
Asceticism T1	−0.1	−1.72	0.03	0.25	−0.13	−1.81
Impulse Regulation T1	0.14	2.41 *	0.17	1.6	0.09	1.3
Social Insecurity T1	0.15	2.27 *	0.15	1.02	0.17	2.2 *
Somatization T1	0.57	12.25 ***	0.5	4.94 ***	0.62	11.32 ***
Sinus-Milieus	−0.06	−1.39	0.01	−0.04	−0.07	−1.45
	*F*_(14, 369)_ = 14.14 ***, *R*^2^ = 0.45		*F*_(14, 97)_ = 4.7 ***, *R*^2^ = 0.4		*F*_(14, 258)_ = 17.57 ***, *R*^2^ = 0.49	

* *p* < 0.05, *** *p* < 0.001.

## Data Availability

The dataset underpinning the conclusions of this research is accessible upon request from the corresponding author, S.B.S.-B. These data are not publicly available to protect the confidentiality of research participants and prevent any potential compromise of their privacy.
